# ﻿Six new species and a new synonym of the mesh-web spider genus *Sudesna* Lehtinen, 1967 (Araneae, Dictynidae) from China

**DOI:** 10.3897/zookeys.1234.145300

**Published:** 2025-04-09

**Authors:** Lu-Yu Wang, Xian-Jin Peng, Zhi-Sheng Zhang

**Affiliations:** 1 Key Laboratory of Eco-environments in Three Gorges Reservoir Region (Ministry of Education), School of Life Sciences, Southwest University, Chongqing 400715, China Southwest University Chongqing China; 2 College of Life Sciences, Hunan Normal University, Changsha 410081, Hunan, China Hunan Normal University Changsha China

**Keywords:** Description, Dictyninae, morphology, taxonomy

## Abstract

Six new species of the genus *Sudesna* are described from South China: *S.cangshan***sp. nov.** (♀, Yunnan), *S.dali***sp. nov.** (♂♀, Yunnan), *S.haiboi***sp. nov.** (♂♀, Yunnan), *S.hainan***sp. nov.** (♂♀, Hainan), *S.shangrila***sp. nov.** (♂♀, Yunnan) and *S.yangi***sp. nov.** (♂♀, Yunnan). *Dictynayongshun* Yin, Bao & Kim, 2001 is proposed here as a junior synonym of *S.hedini* (Schenkel, 1936). Detailed descriptions, photographs, along with illustrations of genital organs, somatic features, and a distribution map of *Sudesna* species in China are provided.

## ﻿Introduction

The mesh-web spider genus *Sudesna* Lehtinen, 1967 was established by Lehtinen (1967) based on the type species, *S.hedini* (Schenkel, 1936) (both sexes), from China and three other species, *S.anaulax* (Simon, 1908) (male only) from Australia, *S.grammica* (Simon, 1893) (female only) from Philippines, and *S.grossa* (Simon, 1906) (female only) from India. These species’ distributions indicate that *Sudesna* may be endemic to Australia, South and Southeast Asia. Additional exploration should be done to find the other sex of the aforementioned three species, and likely more species will also be found. Zhang and Li (2011) described two species from Xishuangbanna, Yunnan, China, a small tropical area, indicating that *Sudesna* has a relatively high diversity in South China. Esyunin and Sozontov (2016) transferred a seventh species to *Sudesna*, *S.flavipes* (Hu, 2001) (female only), after their analysis of the morphological characters of some Palearctic *Dictyna* Sundevall, 1833 species.

Based on newly collected material from China, we propose six new species and suggest a new synonym of the type species, *S.hedini* from Hunan Province. Thus, the genus *Sudesna* contains 13 species from Australia and South, Southeast and East Asia. Except for the type species, which has been recorded from China and South Korea, each *Sudesna* species is known only from their type locality. Our field observations indicate that *Sudesna* species prefer to live in small mesh-webs on the underside of leaves, and they have been collected by beating leaves (Zhang and Wang 2017).

## ﻿Materials and methods

All specimens are preserved in 75% ethanol and were examined, illustrated, photographed, and measured using a Leica M205A stereomicroscope equipped with a drawing tube, a Leica DFC450 Camera, and LAS v. 4.6 software. Male palps and epigynes were examined and illustrated after dissection. Epigynes were cleared by immersing them in a pancreatin solution (Álvarez-Padilla and Hormiga 2007). Eye sizes were measured as the maximum dorsal diameter. Leg measurements are shown as: total length (femur, patella and tibia, metatarsus, tarsus). All measurements are in millimetres. Specimens examined here are deposited in the Collection of Spiders, School of Life Sciences, Southwest University, Chongqing, China (**SWUC**) and in the Hunan Normal University (**HNU**).

Abbreviations used in the text: **ALE**, anterior lateral eye; **AME**, anterior median eye; **MOA**, median ocular area; **PLE**, posterior lateral eye; **PME**, posterior median eye.

## ﻿Taxonomy

### ﻿Family Dictynidae O. Pickard-Cambridge, 1871 (卷叶蛛科)

**Subfamily Dictyninae O. Pickard-Cambridge, 1871** (卷叶蛛亚科)

#### ﻿Genus Sudesna

Taxon classificationAnimaliaAraneaeDictynidae

Lehtinen, 1967

2A5EDB06-DA64-5264-B43E-1F122FF6C4CB

##### Type species.

*Dictynahedini* Schenkel, 1936.

##### Diagnosis.

*Sudesna* can be distinguished from *Dictyna* by the reduced or absent spur of the male palpal tibia, the anteriorly located, widely separated epigynal copulatory openings, the tube-like copulatory ducts, and the laterally located spermathecal heads (Zhang and Li 2011).

##### Description.

Diminutive in size (1.60–4.57). Dorsum of prosoma pale darker to brown, with high cephalic area. Fovea absent. Cervical groove distinct, radial furrows indistinct. Eight eyes in 2 rows; eyes located on eye tubercles; eye tubercles coloured same as carapace. Chelicerae stout, yellowish to brown, with small yellow lateral condyles, 3–4 promarginal and 1–3 retromarginal teeth. Endites yellow, longer than wide. Labium yellow-brown, as long as wide. Sternum yellow-brown, with truncated anterior margin and blunt posterior margin. Legs yellowish to brown, patella with a small protrusion. Opisthosoma oval. Dorsum pale to darker brown, with some small, white, scale-like markings near midline. Venter of abdomen yellow-brown, with small, undivided cribellum. Spinnerets short and yellowish brown.

Palp with droplet-shaped cymbium. Tibia dorsally with two ctenidia or absent (*S.circularis* Zhang & Li, 2011 and *S.yangi* sp. nov.). Retrolateral tibial apophysis triangular or hook-shaped. Embolus semicircular and originating at about 8:00 to 11:30 o’clock position. Anterior arm of conductor (AA) short and membranous; posterior arm (PA) finger-shaped or twisted, usually with scaly tip.

Epigyne with widely separated copulatory openings. Copulatory openings with pronounced inner margins. Copulatory ducts short or long, usually twisted, and membranous or slightly sclerotized. Spermathecae sclerotized, irregularly shaped. Spermathecal heads spherical or oval. Fertilization ducts thin, long, extending from the anterior parts of spermathecae, curving and pointing laterally.

### ﻿Key to species

**Table:** 

1	Male	**2**
–	Female	**7**
2	Tibia without ctenidia	***S.yangi* sp. nov.**
–	Tibia with ctenidia	**3**
3	Posterior arm of conductor straight	***S.dali* sp. nov.**
–	Posterior arm of conductor twisted	**4**
4	Embolus originating at about the 11:30 o’clock position	***S.hainan* sp. nov.**
–	Embolus originating at about the 8:30 to 10:00 o’clock position	**5**
5	Opisthosoma dark	***S.haiboi* sp. nov.**
–	Opisthosoma pale	**6**
6	Posterior arm of the conductor strongly curved	***S.hedini* (Schenkel, 1936)**
–	Posterior arm of the conductor slightly curved	***S.shangrila* sp. nov.**
7	Spermatheca bifurcate	***S.cangshan* sp. nov.**
–	Spermatheca simple	**8**
8	Spermathecal heads oval; spermatheca spiral	***S.haiboi* sp. nov.**
–	Spermathecal heads spherical; spermatheca not spiral	**9**
9	Copulatory ducts short, slightly curved	**10**
–	Copulatory ducts long, strongly curved	**12**
10	Edges of spermatheca tuberculate	***S.dali* sp. nov.**
–	Edges of spermatheca smooth	**11**
11	Diameter of spermathecal heads equal to width of copulatory openings	***S.hainan* sp. nov.**
–	Diameter of spermathecal heads greater than width of copulatory openings	***S.shangrila* sp. nov.**
12	Diameter of spermathecal heads equal to width of spermatheca	***S.yangi* sp. nov.**
–	Diameter of spermathecal heads less than width of spermatheca	***S.hedini* (Schenkel, 1936)**

#### ﻿ Sudesna cangshan sp. nov.

Taxon classificationAnimaliaAraneaeDictynidae

26EBB14F-502A-5E4E-AF0A-4EE3BBC67132

https://zoobank.org/D8657831-FABF-41DE-9754-3350542064DF

**Figure 1.**
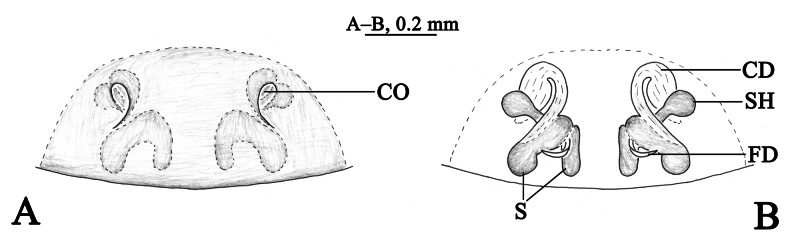
*Sudesnacangshan* sp. nov. holotype female **A** epigyne, ventral view **B** vulva, dorsal view. Abbreviations: CD = copulatory duct; CO = copulatory opening; FD = fertilization duct; S = spermatheca; SH = spermathecal head.

**Figure 2.**
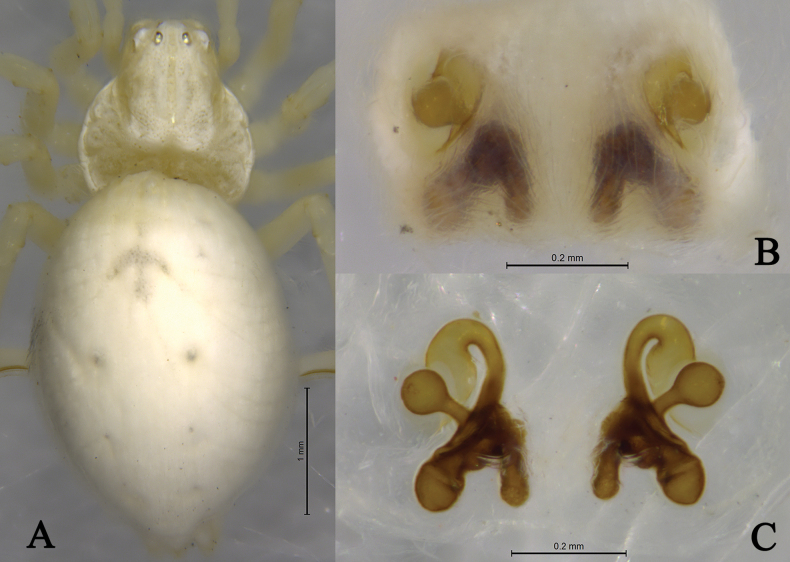
*Sudesnacangshan* sp. nov. holotype female **A** habitus, dorsal view **B** epigyne, ventral view **C** vulva, dorsal view.

Figs 1 , 2 , 15 (苍山苏蛛)

##### Type material.

***Holotype***: China • ♀ (SWUC-T-DI-14-01); Yunnan Province; Dali City, Mount Cangshan, Dapoqing; 25°34′38″N, 100°07′51″E; elev. 2043 m; 30 August 2009; Z.Z. Yang leg.

##### Etymology.

The specific name is derived from the county where the type locality is located; used as a noun in apposition.

##### Diagnosis.

The female of this new species is similar to *S.yangi* sp. nov. (Figs 13, 14) in having a pale body and 9-shaped copulatory openings, but it can be distinguished by having copulatory openings as large as the spermathecal head (vs twice as large), gently curved copulatory ducts (vs abruptly bent at an acute angle), and bifurcate spermatheca (vs clavate) (Figs 1, 2B, C).

##### Description.

Female holotype (Fig. 2A) total length 3.31. Prosoma 1.27 long, 1.16 wide; Opisthosoma 2.23 long, 1.65 wide. Dorsum of prosoma pale, with high cephalic area. Eye sizes and interdistances: AME 0.05, ALE 0.08, PME 0.08, PLE 0.07; AME–AME 0.11, AME–ALE 0.09, PME–PME 0.13, PME–PLE 0.12, ALE–PLE 0.02. MOA 0.20 long, anterior width 0.20, posterior width 0.27. Clypeus height 0.12. Chelicerae stout, yellowish brown, with 3 promarginal and 3 retromarginal teeth. Legs yellowish. Leg measurements: I 3.73 (1.04, 1.58, 0.70, 0.41); II 3.28 (1.04, 1.17, 0.68, 0.39), III 2.72 (0.92, 0.91, 0.58, 0.31), IV 3.20 (1.02, 1.12, 0.75, 0.31). Leg formula: 1243. Opisthosoma oval. Dorsum and venter pale. Spinnerets short and yellowish brown.

***Epigyne*** (Figs 1, 2B, C). Copulatory openings somewhat 9-shaped and facing away from each other, separated by about 6 times their width. Copulatory ducts membranous, gently curved, longer than spermathecal length. Spermatheca bifurcate, spermathecal heads spherical. Fertilization ducts thin, curved, directed laterally.

Male unknown.

##### Distribution.

Known only from the type locality, Yunnan, China (Fig. 15).

#### ﻿ Sudesna dali sp. nov.

Taxon classificationAnimaliaAraneaeDictynidae

2D4F6880-5E9E-50F1-AD91-58A02A6BFB29

https://zoobank.org/CE4698DA-3BB0-4BFF-8815-C8C8F84B0AF9

**Figure 3.**
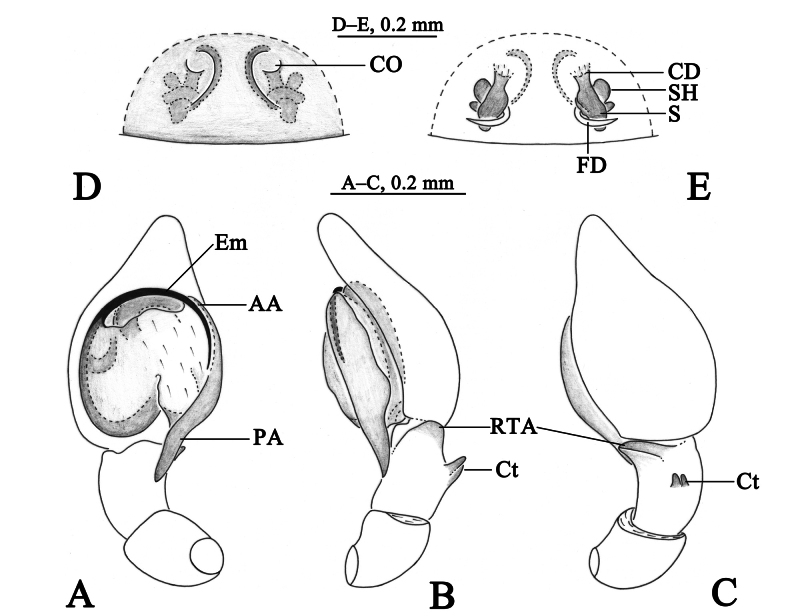
*Sudesnadali* sp. nov. holotype male (**A–C**) and paratype female (**D, E**). **A** Left male palp, ventral view **B** same, retrolateral view **C** same, dorsal view **D** epigyne, ventral view **E** vulva, dorsal view. Abbreviations: AA = anterior arm of conductor; CD = copulatory duct; CO = copulatory opening; Ct = ctenidia; Em = embolus; FD = fertilization duct; PA = posterior arm of conductor; RTA = retrolateral tibial apophysis; S = spermathecal; SH = spermathecal head.

**Figure 4.**
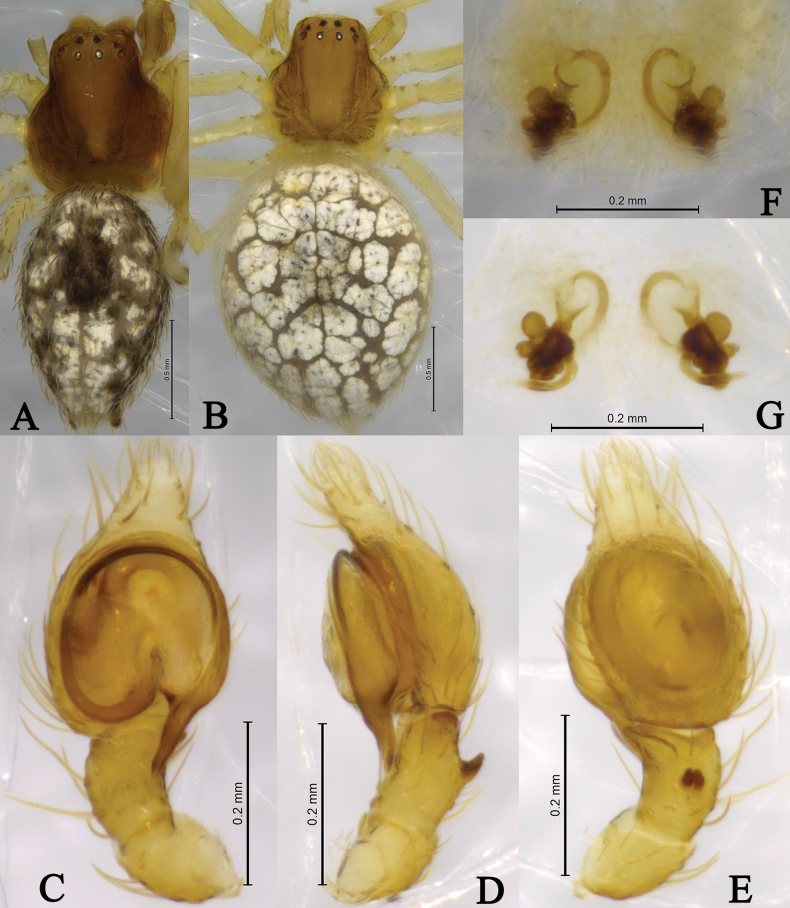
*Sudesnadali* sp. nov. holotype male (**A, C–E**) and paratype female (**B, F, G**). **A** Male habitus, dorsal view **B** female habitus, dorsal view **C** left male palp, ventral view **D** same, retrolateral view **E** same, dorsal view **F** epigyne, ventral view **G** vulva, dorsal view.

Figs 3 , 4 , 15 (大理苏蛛)

##### Type material.

***Holotype*** ♂ (SWUC-T-DI-15-01); **Yunnan Province**; Dali City, Mount Cangshan, Dapoqing; 25°34′59″N, 100°08′09″E; elev. 2500 m; 8 November 2009; T.B. Yang leg. ***Paratypes***: 1 ♂ 3 ♀ (SWUC-T-DI-15-02 to 05); same data as for holotype • 3 ♂ 2 ♀ (SWUC-T-DI-15-06 to 10); Mount Cangshan, Yujufeng; 25°42′14″N, 100°07′09″E; elev. 2700 m; 10 April 2011; L. Yang leg. • 2 ♀ (SWUC-T-DI-15-11 to 12); Mount Cangshan; elev. 2500 m; 15 January 2009; Z.Z. Yang leg. • 4 ♂ 16 ♀ (SWUC-T-DI-15-13 to 32); Mount Cangshan, Xieyangfeng; 25°36′04″N, 100°11′11″E; elev. 2559 m; 14 June 2011; K.C. Zhang leg. • 1 ♂ (SWUC-T-DI-15-33); Mount Cangshan, Xieyangfeng; 25°35′54″N, 100°11′13″E; elev. 2700 m; 14 June 2011; Z.X. Bao leg. • 1 ♂ (SWUC-T-DI-15-34); Mount Cangshan; Xieyangfeng; 25°35′58″N, 100°11′7″E; elev. 2500 m; 10 April 2011; N.J. Li leg. • 1 ♂ 1 ♀ (SWUC-T-DI-15-35 to 36); Mount Cangshan, Xieyangfeng; 25°35′54″N, 100°11′13″E; elev. 2615 m; 15 January 2009; Z.Z. Yang leg.

##### Etymology.

The specific name is derived from the county where the type locality is located; used as a noun in apposition.

##### Diagnosis.

This new species is similar to *S.hainan* sp. nov. (Figs 7, 8) in having a semicircular embolus and short copulatory ducts, but it can be distinguished by the triangular (dorsal view) retrolateral tibial apophysis (vs semicircular, lamellate), the finger-shaped and untwisted posterior arm (PA) of the conductor (vs somewhat S-shaped) (Figs 3A–C, 4C–E), and the absence of a stem of the spermathecal head (vs present) (Figs 3E, 4G).

##### Description.

**Male holotype** (Fig. 4A) total length 2.00. Prosoma 0.86 long, 0.78 wide; Opisthosoma 1.20 long, 0.75 wide. Dorsum of prosoma brown, with high cephalic area. Eye sizes and interdistances: AME 0.03, ALE 0.04, PME 0.04, PLE, 0.04; AME–AME 0.09, AME–ALE 0.06, PME–PME 0.08, PME–PLE 0.10, ALE–PLE 0.02. MOA 0.14 long, anterior width 0.14, posterior width 0.16. Clypeus height 0.09. Chelicerae stout, yellow-brown, with 3 promarginal teeth and 1 retromarginal tooth. Legs yellowish. Leg measurements: I 2.76 (0.84, 0.95, 0.59, 0.38); II 2.56 (0.78, 0.88, 0.50, 0.40); III 2.03 (0.62, 0.65, 0.46, 0.30); IV 2.28 (0.73, 0.69, 0.55, 0.31). Leg formula: 1243. Opisthosoma oval. Dorsum with black and white markings. Venter yellow brown. Spinnerets short and yellow-brown, with black markings.

***Palp*** (Figs 3A, B, 4C–E). Tibia dorsally with 2 ctenidia, located at 1/3 length of tibia from the distal-most part. Retrolateral tibial apophysis triangular in dorsal view. Embolus semicircular and originating at about the 10:00 o’clock position and terminating at about the 4:30 o’clock position. Anterior arm of conductor (AA) short and membranous; posterior arm (PA) finger-shaped with sharply pointed tip.

**Female paratype** (SWUC-T-DI-15-02, Fig. 4B) total length 2.46. Prosoma 0.86 long, 0.81 wide; opisthosoma 1.64 long, 1.30 wide. Dorsum of prosoma yellow-brown, with high cephalic area. Eye sizes and interdistances: AME 0.04, ALE 0.04, PME 0.04, PLE, 0.04; AME–AME 0.07, AME–ALE 0.06, PME–PME 0.08, PME–PLE 0.08, ALE–PLE 0.02. MOA 0.13 long, anterior width 0.14, posterior width 0.16. Clypeus height 0.06. Leg measurements: I 2.41 (0.75, 0.81, 0.49, 0.36); II 2.32 (0.71, 0.79, 0.48, 0.34); III 1.98 (0.62, 0.63, 0.46, 0.27); IV 2.29 (0.72, 0.78, 0.51, 0.28). Legs yellowish. Leg formula: 1243. Opisthosoma oval. Dorsum yellow-brown, with white scaly markings. Venter yellow-brown. Spinnerets short and yellow brown.

***Epigyne*** (Figs 3C, D, 4F, G). Copulatory openings semicircular, facing each other, separated by about 4 times their width. Copulatory ducts membranous, short, as long as width. Spermatheca peanut-shaped, spermathecal heads somewhat ball-shaped. Fertilization ducts semicircular, directed laterally, the length is 2.5 times the width of the copulatory openings.

##### Variation.

Male (*n* = 12) total length 1.87–2.13; female (*n* = 24) total length 2.18–2.71.

##### Distribution.

Known only from the type locality, Yunnan, China (Fig. 14).

#### ﻿ Sudesna haiboi sp. nov.

Taxon classificationAnimaliaAraneaeDictynidae

69E55D03-0E91-5B6A-87DD-ADA2F124EF4E

https://zoobank.org/823D83E1-E618-4AB5-9A32-DD970521E5C4

**Figure 5.**
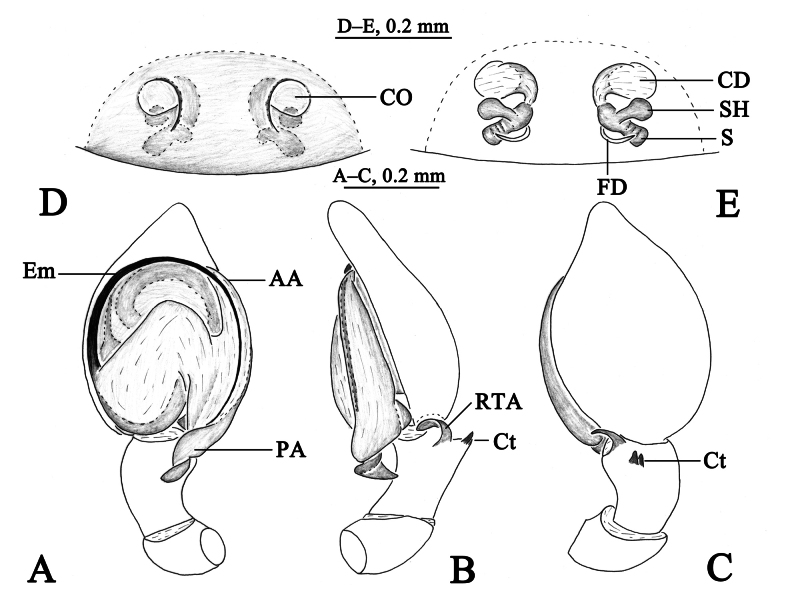
*Sudesnahaiboi* sp. nov. holotype male (**A–C**) and paratype female (**D, E**). **A** Left male palp, ventral view **B** same, retrolateral view **C** same, dorsal view **D** epigyne, ventral view **E** vulva, dorsal view. Abbreviations: AA = anterior arm of conductor; CD = copulatory duct; CO = copulatory opening; Ct = ctenidia; Em = embolus; FD = fertilization duct; PA = posterior arm of conductor; RTA = retrolateral tibial apophysis; S = spermathecal; SH = spermathecal head.

**Figure 6.**
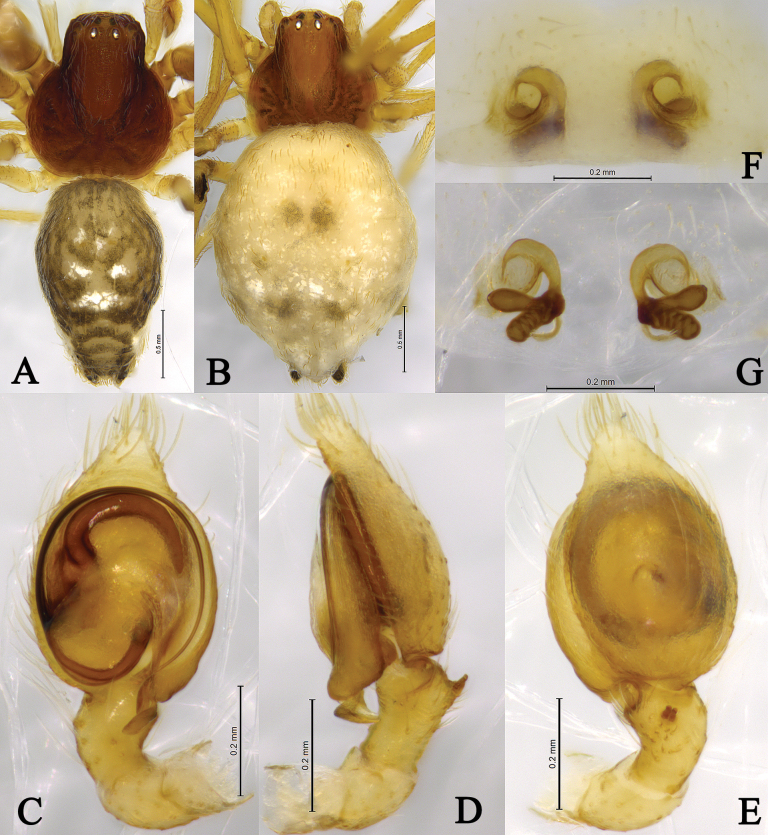
*Sudesnahaiboi* sp. nov. holotype male (**A, C–E**) and paratype female (**B, F, G**). **A** Male habitus, dorsal view **B** female habitus, dorsal view **C** left male palp, ventral view **D** same, retrolateral view **E** same, dorsal view **F** epigyne, ventral view **G** vulva, dorsal view.

Figs 5 , 6 , 15 (海波苏蛛)

##### Type materials.

***Holotype*** ♂ (SWUC-T-DI-16-01); **Yunnan Province**; Dali City, Mount Cangshan, Southern Slopes; 25°34′27″N, 100°7′49″E; elev. 2320 m; May 2010; H.B. Pu and Z.Z. Yang leg. ***Paratypes***: 9 ♂ 10 ♀ (SWUC-T-DI-16-02 to 20); same data as holotype.

##### Etymology.

The specific name honours Mr Haibo Pu, who was of tremendous assistance in the field; a noun in genitive case.

##### Diagnosis.

This species is similar to *S.shangrila* sp. nov. (Figs 11, 12) in having a hook-shaped retrolateral tibial apophysis, a long anterior arm of conductor, and cylindrical spermatheca, but it can be distinguished by the embolus originating at about the 8:30 o’clock position (vs 9:30), the lack of a scaly tip on the posterior arm of the conductor (vs present) (Figs 5A–C, 6C–E), and long, curved copulatory ducts (vs short and straight) (Figs 5E, 6G).

##### Description.

**Male holotype** (Fig. 6A) total length 2.01. Prosoma 0.94 long, 0.87 wide; opisthosoma 1.14 long, 0.76 wide. Dorsum of prosoma brown, with high cephalic area. Eye sizes and interdistances: AME 0.05, ALE 0.05, PME 0.06, PLE, 0.07; AME–AME 0.08, AME–ALE 0.07, PME–PME 0.09, PME–PLE 0.08, ALE–PLE 0.01. MOA 0.16 long, anterior width 0.16, posterior width 0.19. Clypeus height 0.12. Chelicerae stout, brown, with 3 promarginal teeth and 1 retromarginal tooth. Legs yellow-brown. Leg measurements: I 3.45 (1.09, 1.12, 0.80, 0.44), II 3.34 (1.05, 1.08, 0.78, 0.44), III 2.51 (0.79, 0.77, 0.60, 0.35), IV 2.72 (0.83, 0.87, 0.67, 0.35). Leg formula: 1243. Opisthosoma oval. Dorsum yellowish brown, with black and white markings. Venter yellow-brown. Spinnerets short and yellow-brown, with black markings.

***Palp*** (Figs 5A, B, 6C–E). Tibia dorsally with two ctenidia, located 1/3 from distal-most portion of tibia. Retrolateral tibia apophysis hook-shaped. Embolus somewhat O-shaped, originating at about the 8:30 o’clock position and terminating at about the 5:30 o’clock position. Anterior arm of conductor (AA) tapering and terminating at about the 1:00 o’clock position; posterior arm (PA) spiral, with blunt tip.

**Female paratype** (SWUC-T-DI-16-02, Fig. 6B) total length 2.27. Prosoma 0.82 long, 0.83 wide; opisthosoma 1.54 long, 1.27 wide. Dorsum of prosoma yellow-brown, with high cephalic area. Eye sizes and interdistances: AME 0.04, ALE 0.06, PME 0.05, PLE 0.05; AME–AME 0.08, AME–ALE 0.05, PME–PME 0.08, PME–PLE 0.09, ALE–PLE 0.02. MOA 0.14 long, anterior width 0.14, posterior width 0.16. Clypeus height 0.06. Legs yellow-brown. Leg measurements: I 2.96 (0.95, 0.95, 0.67, 0.39), II 2.86 (0.92, 0.92, 0.64, 0.38), III 2.39 (0.77, 0.75, 0.55, 0.32), IV 2.62 (0.82, 0.86, 0.63, 0.31). Leg formula: 1243. Opisthosoma oval. Dorsum yellowish brown, with a few black markings and lots of white scaly markings. Venter yellowish brown. Spinnerets short and yellow-brown, with black markings.

***Epigyne*** (Figs 5C, D, 6F, G). Copulatory openings nearly 9-shaped, facing away from each other, separated by about 2.5 times their width. Copulatory ducts long, membranous, semicircular. Spermatheca spiral; spermathecal heads oval. Fertilization ducts semicircular, laterally directed and as long as width of copulatory openings.

##### Variation.

Male (*n* = 10) total length 1.92–2.10; female (*n* = 10) total length 2.15–2.35.

##### Distribution.

Known only from the type locality, Yunnan, China (Fig. 15).

#### ﻿ Sudesna hainan sp. nov.

Taxon classificationAnimaliaAraneaeDictynidae

069FF572-F123-5430-A399-95C6C98D040C

https://zoobank.org/C7C6A794-7C79-4A8D-85CF-A17C4EF6694D

**Figure 7.**
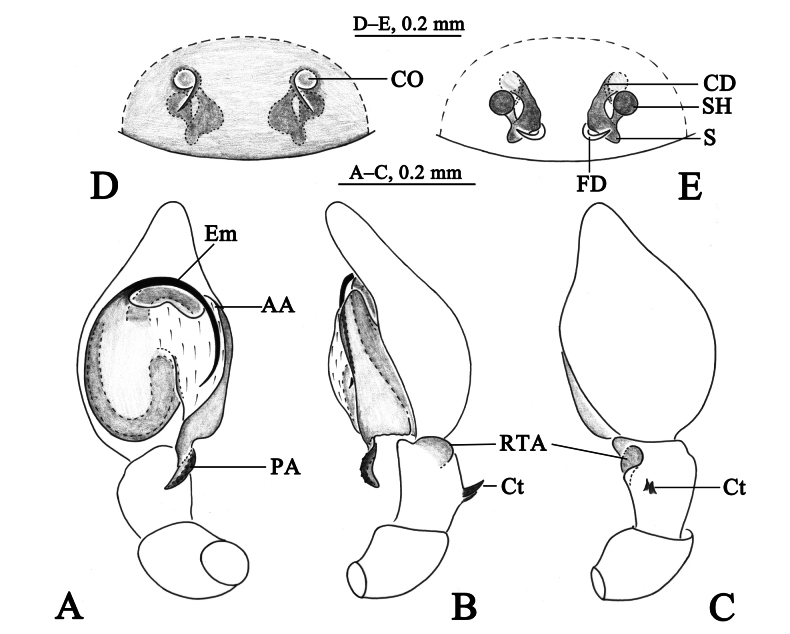
*Sudesnahainan* sp. nov. holotype male (**A–C**) and paratype female (**D, E**). **A** Left male palp, ventral view **B** same, retrolateral view **C** same, dorsal view **D** epigyne, ventral view **E** vulva, dorsal view. Abbreviations: AA = anterior arm of conductor; CD = copulatory duct; CO = copulatory opening; Ct = ctenidia; Em = embolus; FD = fertilization duct; PA = posterior arm of conductor; RTA = retrolateral tibial apophysis; S = spermathecal; SH = spermathecal head.

**Figure 8.**
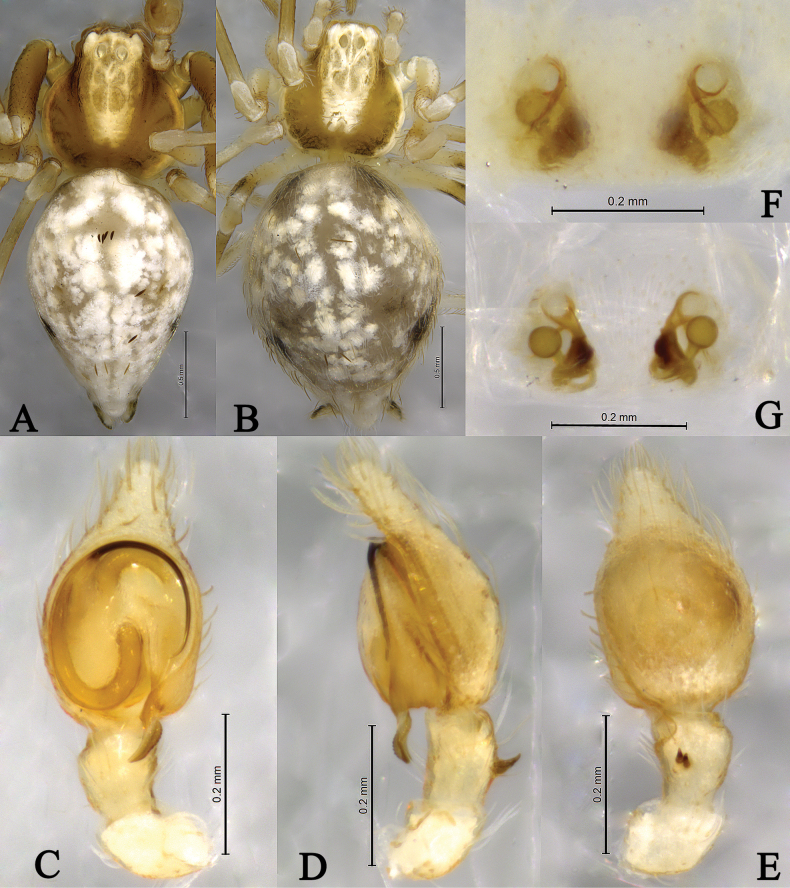
*Sudesnahainan* sp. nov. holotype male (**A, C–E**) and paratype female (**B, F, G**). **A** Male habitus, dorsal view **B** female habitus, dorsal view **C** left male palp, ventral view **D** same, retrolateral view **E** same, dorsal view **F** epigyne, ventral view **G** vulva, dorsal view.

Figs 7 , 8 , 15 (海南苏蛛)

##### Type materials.

***Holotype*** ♂ (SWUC-T-DI-17-01); **Hainan Province**; Changjiang County, Bawangling; 19°07′51″N, 109°03′22″E; elev. 739 m; 21 May 2009; G.X. Han leg. ***Paratypes***: 1 ♂ 13 ♀ (SWUC-T-DI-17-02 to 15); same data as holotype • 2 ♀ (SWUC-T-DI-17-16 to 17); Bawangling, Yajia; 19°04′46″N, 109°07′35″E; elev. 624 m; 19 May 2009; G.X. Han leg. • 2 ♀ (SWUC-T-DI-17-18 to 19); Diaoluoshan National Nature Reserve; 18°40′08″N, 109°53′53″E; elev. 2225 m; June 2009; G.X. Han leg. • 3 ♀ (SWUC-T-DI-17-20 to 22); Jianfengling National Nature Reserve, Zhufeng; 18°42′35″N, 108°52′35″E; elev. 960 m; 31 May 20009; G.X. Han leg. • 5 ♀ (SWUC-T-DI-17-23 to 27); Jianfengling National Nature Reserve, Tianchi; 18°44′38″N, 108°51′43″E; elev. 811 m; 29 May 20009; G.X. Han leg. • 2 ♂ 7 ♀ (SWUC-T-DI-17-28 to 36); Jianfengling National Nature Reserve, Yulingu; 18°44′59″N, 108°55′16″E; elev. 680 m; 1 June 2009; G.X. Han leg. • 2 ♂ 4 ♀ (SWUC-T-DI-17-37 to 42); Tunchang County, Xichang Town; 19°25′55″N, 110°2′48″E; elev. 124 m; 11 June 2009; G.X. Han leg. • 1 ♂ 1 ♀ (SWUC-T-DI-17-43 to 44); Wuzhishan City, Shuiman Township, Wuzhishan National Nature Reserve; 18°54′23″N, 109°40′51″E; elev. 747 m; Q.L. Lu leg.

##### Etymology.

The specific name is derived from the county where the type locality is located; used as a noun in apposition.

##### Diagnosis.

See the diagnosis of *S.dali* sp. nov.

##### Description.

**Male holotype** (Fig. 8A) total length 2.30. Prosoma 0.91 long, 0.84 wide; opisthosoma 1.48 long, 0.98 wide. Dorsum of prosoma brown, with white high cephalic area. Eye sizes and interdistances: AME 0.04, ALE 0.10, PME 0.08, PLE, 0.09; AME–AME 0.07, AME–ALE 0.04, PME–PME 0.06, PME–PLE 0.10, ALE–PLE 0.02. MOA 0.20 long, anterior width 0.15, posterior width 0.22. Clypeus height 0.04. Chelicerae stout, brown, with 3 promarginal teeth and 1 retromarginal tooth. Legs yellow-brown (femur I brown). Leg measurements: I 3.27 (1.04, 1.16, 0.62, 0.45); II 3.05 (1.03, 1.02, 0.57, 0.43); III 2.35 (0.75, 0.74, 0.50, 0.36); IV 2.59 (0.81, 0.85, 0.54, 0.39). Leg formula: 1243. Opisthosoma almost pyriform. Dorsum white, with a few short, strong spines. Venter yellowish brown. Spinnerets short and yellowish brown with black markings.

***Palp*** (Figs 7A, B, 8C–E). Tibia dorsally with 2 ctenidia, located at middle. Retrolateral tibia apophysis lamellate. Embolus semicircular and originating at about the 11:30 o’clock position, terminating at about the 4:00 o’clock position. Anterior arm of conductor (AA) membranous, terminating at about the 1:30 o’clock position; posterior arm (PA) backwards S-shaped in retrolateral view, with a blunt, scaly tip.

**Female paratype** (SWUC-T-DI-17-02, Fig. 8B) total length 2.42. Prosoma 0.85 long, 0.79 wide; opisthosoma 1.58 long, 1.24 wide. Dorsum of prosoma yellowish brown, with white high cephalic area. Eye sizes and interdistances: AME 0.03, ALE 0.08, PME 0.07, PLE, 0.08; AME–AME 0.06, AME–ALE 0.03, PME–PME 0.05, PME–PLE 0.07, ALE–PLE 0.02. MOA 0.17 long, anterior width 0.11, posterior width 0.17. Clypeus height 0.04. Legs yellowish brown, with black markings. Leg measurements: I 2.87 (0.87, 1.00, 0.60, 0.40); II 2.85 (0.93, 0.94, 0.57, 0.41); III 2.25 (0.70, 0.70, 0.51, 0.34); IV 2.55 (0.77, 0.84, 0.54, 0.40). Leg formula: 1243. Opisthosoma oval. Dorsum yellowish brown, with a few black markings and many white scaly markings. Venter yellowish brown. Spinnerets short and yellow-brown with black markings.

***Epigyne*** (Figs 7C, D, 8F, G). Copulatory openings nearly 9-shaped, facing away from each other, separated by about 4.5 times their width. Copulatory ducts slightly sclerotized, short, slightly curved. Spermatheca curved, spermathecal heads round. Fertilization ducts thin, semicircular, and directed laterally.

##### Variation.

Male (*n* = 5) total length 1.91–2.30; female (*n* = 37) total length 2.24–2.42.

##### Distribution.

China (Hainan) (Fig. 15).

#### ﻿ Sudesna hedini

Taxon classificationAnimaliaAraneaeDictynidae

(Schenkel, 1936)

A09EEF84-3C34-539F-8BFB-CF31DBF52A84

**Figure 9.**
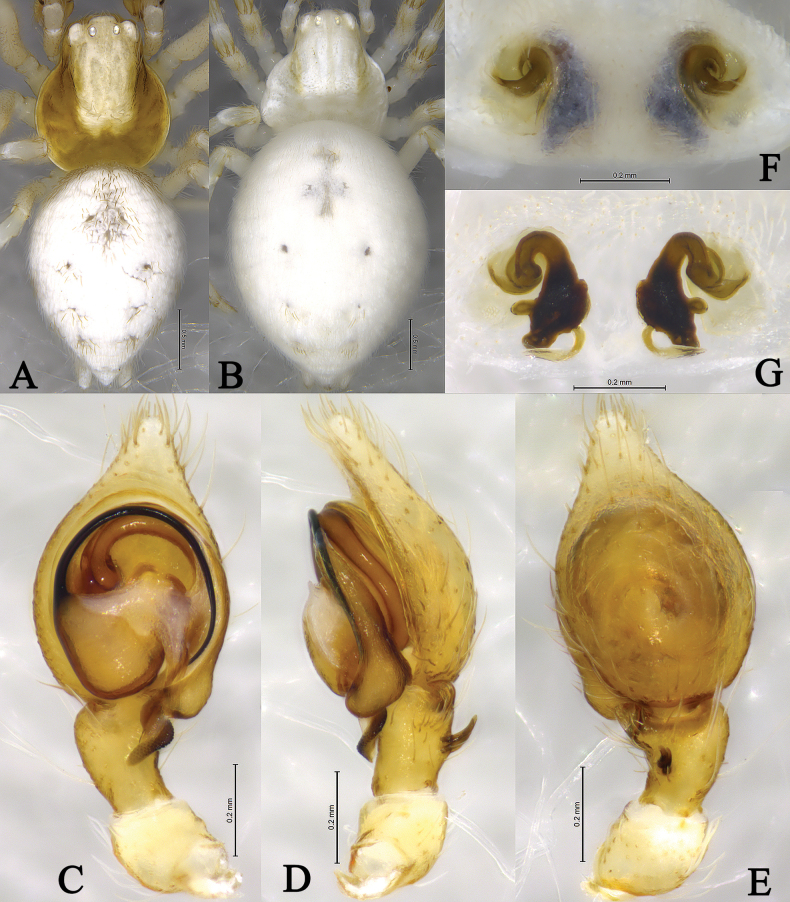
*Sudesnahedini* (Schenkel, 1936) male (SWUC-DI-SH-56, **A, C–E**) and female (SWUC-DI-SH-57, **B, F, G**). **A** Male habitus, dorsal view **B** female habitus, dorsal view **C** left male palp, ventral view **D** same, retrolateral view **E** same, dorsal view **F** epigyne, ventral view **G** vulva, dorsal view.

**Figure 10.**
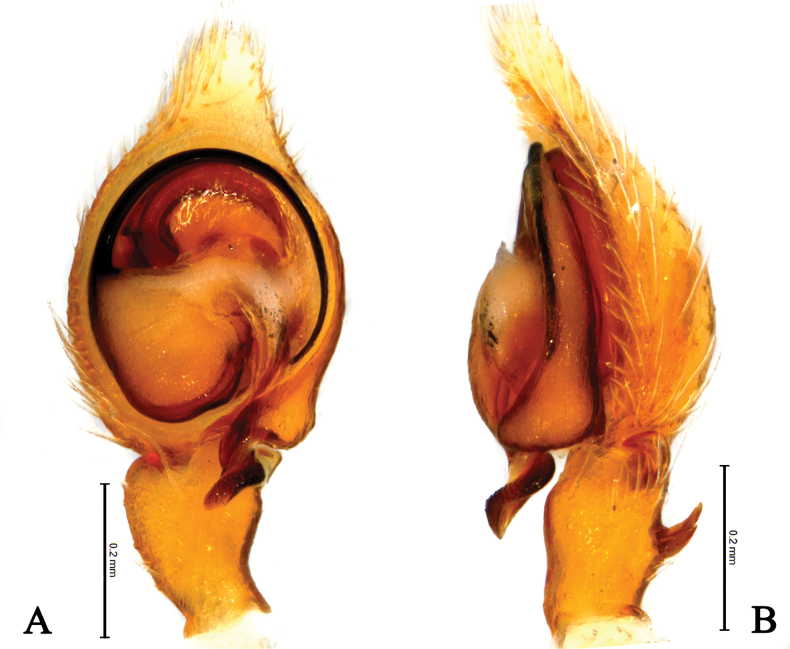
*Sudesnahedini* (Schenkel, 1936) (holotype of *Dictynayongshun* Yin, Bao & Kim, 2001). **A** Left male palp, ventral view **B** same, retrolateral view.

Figs 9 , 10 , 15 (赫氏苏蛛)

Dictyna hedini Schenkel, 1936: 14, f. 2 (♂♀); Paik 1979: 423, figs 11–21 (♂♀); Zhu and Shi 1985: 57, figs 46a–c (♀).
Sudesna hedini : Lehtinen, 1967: 265, figs 305, 318 (♂♀); Song and Lu 1985: 80, fig. 4A, B (♀); Song 1987: 79, fig. 43 (♀); Feng 1990: 37, fig. 12 (♀); Song et al. 1999: 365, fig. 216D, E (♀); Song et al. 2001: 288, fig. 182A, B (♀); Namkung 2002: 385, fig. 27.10a, b (♂♀); Namkung 2003: 387, fig. 27.10a, b (♂♀); Marusik et al. 2006: 355, fig. 5 (♂); Zhang and Li 2011: 30, fig. 7A–H (♂♀); Esyunin and Sozontov 2016: 204, fig. 13 (♀); Kim and Lee 2017: 49, fig. 28A, B (♀).
Dictyna yongshun Yin, Bao & Kim, 2001: 170, figs 1–4 (♂); Yin et al. 2012: 976, fig. 496a–d (♂). syn. nov.

##### Material examined.

**Hunan**: 1 ♂ (**holotype** of *Dictynayongshun* Yin, Bao & Kim, 2001), Yongshun County, Buermen; 12 September 1996; C.M. Yin leg. (HNU) • **Guizhou**: 1 ♀ (SWUC-DI-SH-01); Guiyang City, Qianling Park; 26°35′55″N, 106°41′35″E; elev. 1172 m; 8 October 2012; L.Y. Wang, X.K. Jiang leg. • 1 ♂ (SWUC-DI-SH-02), Guiding County, Yanxia Town; 26°23′00″N, 107°18′13″E; elev. 1173 m; 8 August 2007; Z.S. Zhang leg; • 2 ♂ (SWUC-DI-SH-03 to 04); Xishiu County, Tucheng Town; 28°16′40″N, 105°59′48″E; elev. 307 m; 17 September 2016; Z.Z. Yang leg. • 6 ♀ (SWUC-DI-SH-05 to 10); Kaili City, Leishan County, Leigongshan National Nature Reserve, Xiaodanjiang; 26°21′21″N, 108°09′30″E; elev. 1409 m; 17 September 2005; Z.S. Zhang, H.M. Chen leg. • **Hebei**: 1 ♀ (SWUC-DI-SH-11); Xiaowutai Mountain, Yangjiaping, 39°56'10"N, 114°56'43"E; elev. 1440 m; 30 July 2012; F. Zhang leg. • **Hubei**: 1 ♀ (SWUC-DI-SH-12); Shennongjia, Muyuping; 31°27′54″N, 110°23′53″E; elev. 1207m; 24 September 2001; M.S. Zhu leg. • **Liaoning**: 1 ♂ (SWUC-DI-SH-13); Dandong City, Jinjiangshan Park; 40°08′02″N, 124°22′32″E; elev. 84 m; 16 August 2009; H.M. Chen, Z. Li, H.P. Wang leg. • 1 ♀ (SWUC-DI-SH-14); Benxi City, Huanren County, Wunvshan; 41°19′38″N, 125°25′01″E; elev. 627 m; 21 August 2009; H.M. Chen, Z. Li, H.P. Wang leg. • 4 ♂ 14 ♀ (SWUC-DI-SH-15 to 32); Benxi City, Huanren County, Wangtiandong Scenic Spot; 41°11′24″N, 125°16′04″E; elev. 280 m; 20 August 2009; H.M. Chen, Z. Li, H.P. Wang leg. • 1 ♂ (SWUC-DI-SH-33); Kuandian County, Tianhuashan Scenic Spot; 41°05′06″N, 124°34′50″E; elev. 724 m; 17 August 2009; H.M. Chen, Z. Li, H.P. Wang leg. • **Shaanxi**: 1 ♂ (SWUC-DI-SH-34); Mei County, Tangyu, Taibaishan; 34°07′46″N, 107°53′50″E; elev. 703 m; 10 September 2004; Z.S. Zhang, H.M. Chen leg. • 2 ♀ (SWUC-DI-SH-35 to 36); Zhouzhi County, Houzhenzi Town; 33°50′49″N, 107°50′01″E; elev. 1305 m; 14 September 2011; L.Y. Wang, Z. Li, D. Wang leg. • **Sichuan**: 8 ♂ 11 ♀ (SWUC-DI-SH-37 to 55); Jiulong County, Wanba Town; 29°04′16″N, 102°03′09″E; elev. 2078 m; 28 September 2008; H.M. Chen leg. • 1 ♂ 2 ♀ (SWUC-DI-SH-56 to 58); Luding County, Moxi Town; 29°39′04″N, 102°06′53″E; elev. 1572 m; 12 November 2019; Z.S. Zhang, L.Y. Wang leg.

##### Diagnosis.

**Figure 11.**
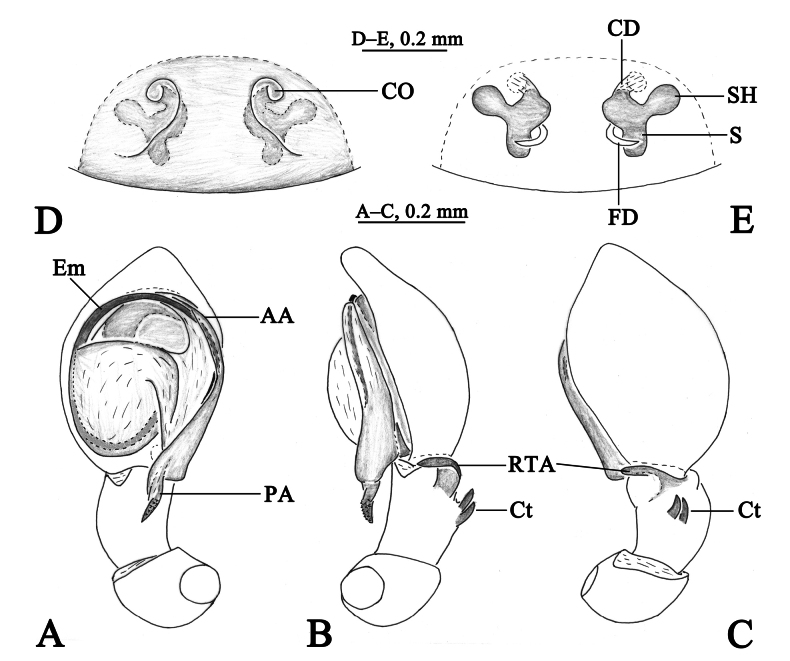
*Sudesnashangrila* sp. nov. holotype male (**A–C**) and paratype female (**D, E**). **A** Left male palp, ventral view **B** same, retrolateral view **C** same, dorsal view **D** epigyne, ventral view **E** vulva, dorsal view. Abbreviations: AA = anterior arm of conductor; CD = copulatory duct; CO = copulatory opening; Ct = ctenidia; Em = embolus; FD = fertilization duct; PA = posterior arm of conductor; RTA = retrolateral tibial apophysis; S = spermathecal; SH = spermathecal head.

**Figure 12.**
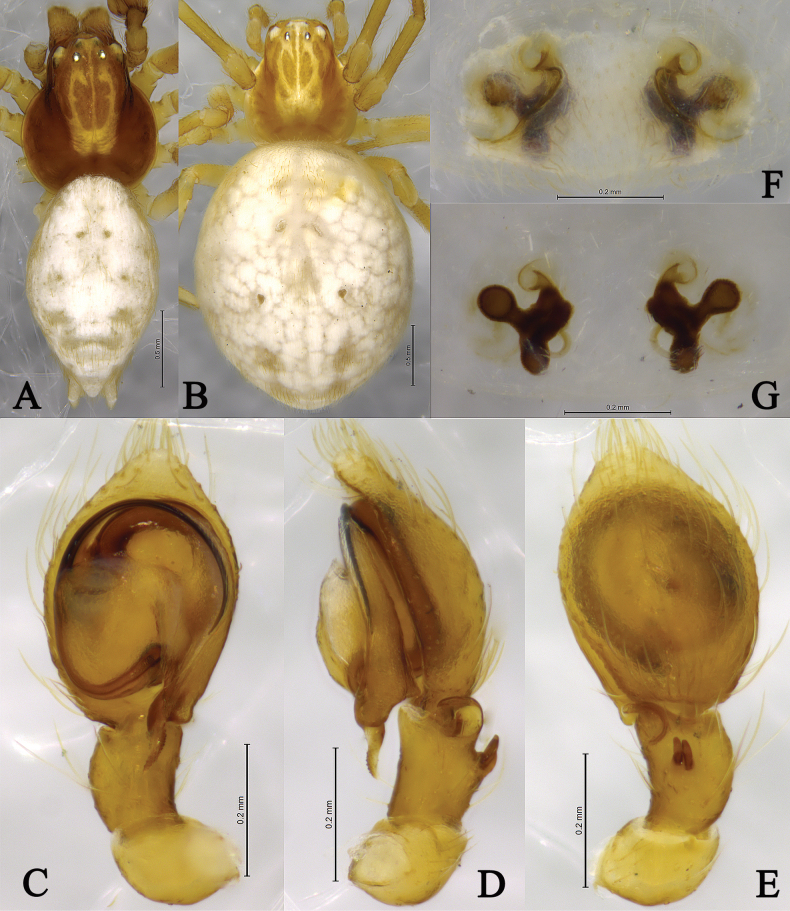
*Sudesnashangrila* sp. nov. holotype male (**A, C–E**) and paratype female (**B, F, G**). **A** Male habitus, dorsal view **B** female habitus, dorsal view **C** left male palp, ventral view **D** same, retrolateral view **E** same, dorsal view **F** epigyne, ventral view **G** vulva, dorsal view.

This species is similar to *S.shangrila* sp. nov. (Figs 11, 12) in having a hook-shaped retrolateral tibial apophysis and spiraled copulatory openings, but it can be distinguished by the spiral posterior arm of the conductor (vs slightly twisted), the curved copulatory duct (vs columnar), and the bud-shaped spermathecal head (vs round).

##### Distribution.

China (Beijing, Gansu, Guizhou, Hebei, Hubei, Hunan, Liaoning, Shanxi, Shaanxi, Sichuan, Zhejiang) (Fig. 15).

##### Remarks.

Although we did not examine the type of *S.hedini*, the published figures of it (Schenkel 1936; Lehtinen 1967) are clear enough. We carefully compared *D.yongshun* (type examined, Fig. 10) and *S.hedini* (Fig. 9), and found that these two species share the same structures, such as the helical posterior arm of conductor, the semicircular embolus originating at about the 9:00 o’clock position, and two ctenidia located dorso-medially on the tibia. Therefore, we consider *D.yongshun* to be a junior synonym of *S.hedini* (Schenkel, 1936).

#### ﻿ Sudesna shangrila sp. nov.

Taxon classificationAnimaliaAraneaeDictynidae

79437C99-F131-5A7F-A391-620074788C38

https://zoobank.org/6CB767CD-9CBC-4E37-B45E-73F1B9FF087D

Figs 11 , 12 , 15 (香格里拉苏蛛)

##### Type material.

***Holotype*** ♂ (SWUC-T-DI-18-01); **Yunnan Province**; Shangrila, Tianshengqiao, Geothermal park; 27°47′53″N, 99°48′46″E; elev. 3406 m; 21 August 2009; Z.X. Li and L.Y. Wang leg. ***Paratypes***: 1 ♂ 7 ♀, (SWUC-T-DI-18-02 to 09); same data as holotype.

##### Etymology.

The specific name is derived from the county where the type locality is located; used as a noun in apposition.

##### Diagnosis.

See the diagnosis of *S.haiboi* sp. nov.

##### Description.

**Male holotype** (Fig. 12A) total length 2.30. Prosoma 1.04 long, 0.90 wide; opisthosoma 1.38 long, 0.86 wide. Dorsum of prosoma brown, with white high cephalic area. Eye sizes and interdistances: AME 0.05, ALE 0.07, PME 0.05, PLE, 0.07; AME–AME 0.12, AME–ALE 0.08, PME–PME 0.13, PME–PLE 0.13, ALE–PLE 0.03. MOA 0.18 long, anterior width 0.19, posterior width 0.24. Clypeus height 0.11. Chelicerae stout, yellow-brown, with 3 promarginal and 2 retromarginal teeth. Legs yellow brown. Leg measurements: I 2.94 (0.92, 0.99, 0.62, 0.41), II 2.83 (0.87, 0.95, 0.60, 0.41), III 2.25 (0.74, 0.72, 0.48, 0.31), IV 2.49 (0.77, 0.85, 0.58, 0.29). Leg formula: 1243. Opisthosoma oval. Dorsum yellowish brown, with white markings. Venter yellowish brown. Spinnerets short and yellowish brown.

***Palp*** (Figs 11A, B, 12C–E). Tibia dorsally with 2 ctenidia, located 1/3 from distalmost part of tibia. Retrolateral tibia apophysis hook-shaped. Embolus semicircular, originating near the 9:30 o’clock position, terminating at about the 4:00 o’clock position. Anterior arm of conductor (AA) membranous and terminating near the 12:00 o’clock position; posterior arm (PA) slightly twisted with a pointed, scaly tip.

**Female paratype** (SWUC-T-DI-18-02, Fig. 12B) total length 3.76. Prosoma 1.36 long, 1.13 wide; opisthosoma 2.64 long, 1.97 wide. Dorsum of prosoma yellowish brown, with white high cephalic area. Eye sizes and interdistances: AME 0.06, ALE 0.07, PME 0.06, PLE, 0.07; AME–AME 0.16, AME–ALE 0.12, PME–PME 0.17, PME–PLE 0.16, ALE–PLE 0.04. MOA 0.21 long, anterior width 0.25, posterior width 0.29. Clypeus height 0.12. Legs yellowish brown. Leg measurements: I 3.48 (1.09, 1.16, 0.75, 0.48), II 3.47 (1.05, 1.15, 0.70, 0.47), III 2.90 (0.94, 0.98, 0.64, 0.34), IV 3.51 (1.12, 1.25, 0.80, 0.34). Leg formula: 1243. Opisthosoma oval. Dorsum yellowish brown, with white scaly markings. Venter yellowish brown. Spinnerets short and yellowish brown.

***Epigyne*** (Figs 11C, D, 12F, G). Copulatory openings nearly spiral, facing away from each other, separated by about 4 times their width. Copulatory ducts short, slightly sclerotized. Spermatheca cylindrical; spermathecal heads round. Fertilization ducts thin, semicircular, and directed laterally.

##### Variation.

Male (*n* = 2) total length 2.06–2.30; female (*n* = 7) total length 2.65–3.76.

##### Distribution.

Known only from the type locality, Yunnan, China (Fig. 15).

#### ﻿ Sudesna yangi sp. nov.

Taxon classificationAnimaliaAraneaeDictynidae

B4BAEAAC-8D11-5DB7-8B8B-BEEE79863926

https://zoobank.org/8D303C1E-237D-4A68-8EB3-7EED0A0B6413

**Figure 13.**
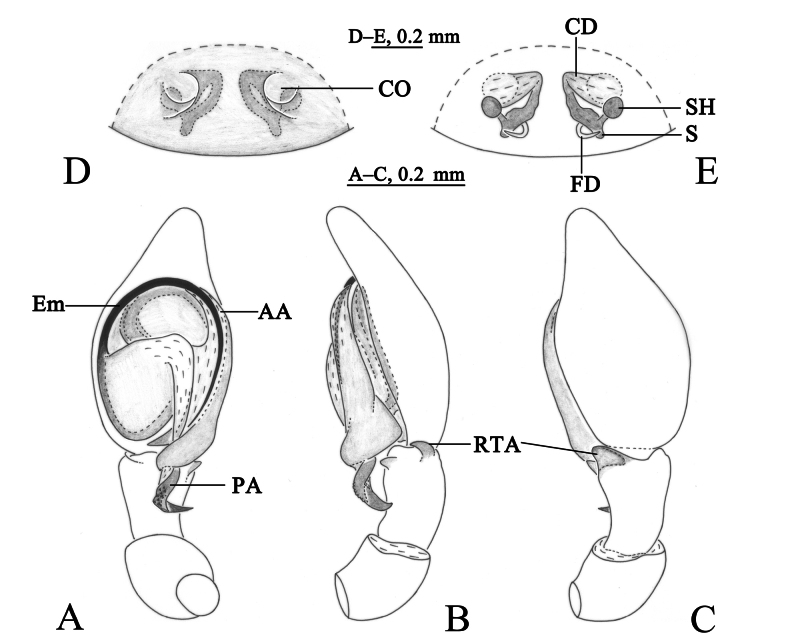
*Sudesnayangi* sp. nov. holotype male (**A–C**) and paratype female (**D, E**). **A** Left male palp, ventral view **B** same, retrolateral view **C** same, dorsal view **D** epigyne, ventral view **E** vulva, dorsal view. Abbreviations: AA = anterior arm of conductor; CD = copulatory duct; CO = copulatory opening; Ct = ctenidia; Em = embolus; FD = fertilization duct; PA = posterior arm of conductor; RTA = retrolateral tibial apophysis; S = spermathecal; SH = spermathecal head.

**Figure 14.**
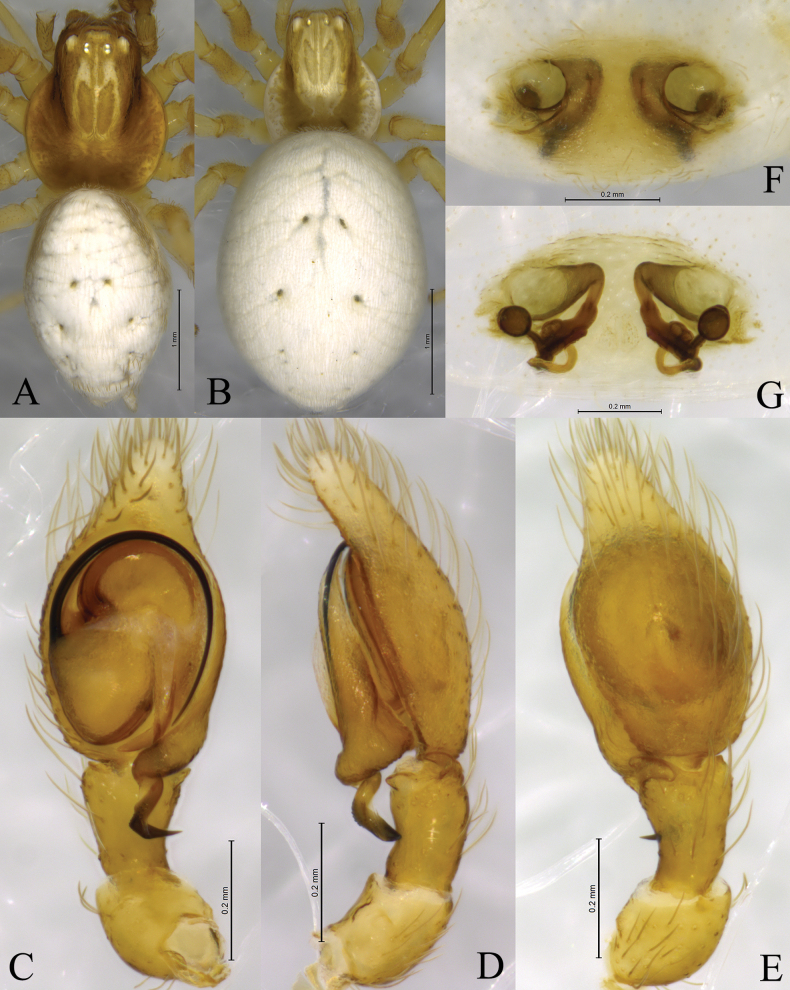
*Sudesnashangrila* sp. nov. holotype male (**A, C–E**) and paratype female (**B, F, G**). **A** Male habitus, dorsal view **B** female habitus, dorsal view **C** left male palp, ventral view **D** same, retrolateral view **E** same, dorsal view **F** epigyne, ventral view **G** vulva, dorsal view.

**Figure 15.**
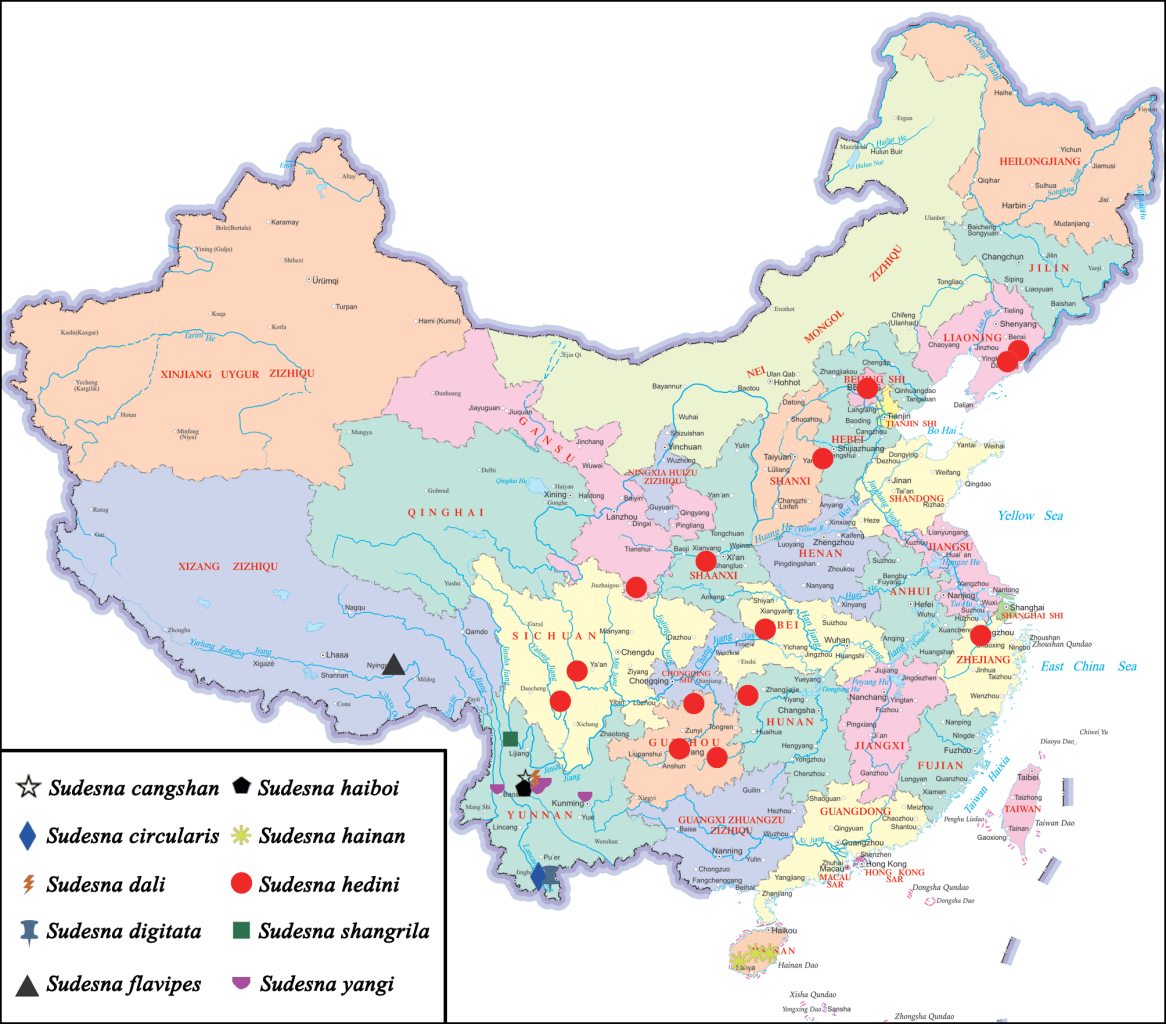
Distribution of *Sudesna* species in China.

Figs 13 , 14 , 15 (杨氏苏蛛)

##### Type material.

***Holotype*** ♂ (SWUC-T-DI-19-01); **Yunnan Province**; Dali City, Mount Cangshan, Xieyangfeng; 25°35′39″N, 100°11′23″E; elev. 2573 m; 11 September 2011; Z.Z. Yang leg. ***Paratypes***: 3 ♂ 6 ♀ (SWUC-T-DI-19-02 to 10); same data as holotype. • 1 ♀ (SWUC-T-DI-19-11); Mount Cangshan, Dapoqing; 25°34′38″N, 100°07′51″E; elev. 2043 m; 30 August 2009; Z.Z. Yang leg. • 6 ♂ 4 ♀ (SWUC-T-DI-19-12 to 21); Mount Cangshan, Yujufeng; 25°42′21″N, 100°7′7″E; elev. 2600 m; 13 September 2009; Z.Z. Yang leg. • 1 ♂ (SWUC-T-DI-19-22); Mount Cangshan, Xieyangfeng; 25°35′24″N, 100°11′47″E; elev. 2500 m; 22 November 2011; P. Feng leg. • 2 ♀ (SWUC-T-DI-19-23 to 24); Mount Cangshan, Xieyangfeng; 5°35′40″N, 100°11′21″E; elev. 2500 m; 14 June 2011; Y. He leg. • 3 ♀ (SWUC-T-DI-19-25 to 27); Eyuan County, Cibi Township, Biaoshan; 26°09′41″N, 99°54′21″E; elev. 2310 m; 17 August 2009; Z.X. Li and L.Y. Wang leg. • 1 ♂ (SWUC-T-DI-19-28); Kunming City, Mount Xishan, Maomaoqing; 24°57′20″N, 102°37′54″E; elev. 2204 m; 13 October 2016; G.Q. Huang, X.B. Guo and Y.C. Wang leg. • 1 ♀ (SWUC-T-DI-19-29); Baoshan City, Longyang District, Mangkuan Township, Mount Gaoligong; 25°18′27″N, 98°47′42″E; elev. 1496 m; 6 October 2015; T. Lu and Y.C. Zhou leg. • 2 ♀ (SWUC-T-DI-19-30 to 31); Baoshan City, Longyang District, Mangkuan Township, Mount Gaoligong; 25°18′05″N, 98°47′24″E; elev. 1793 m; 8 October 2015; T. Lu and Y.C. Zhou leg.

##### Etymology.

This species is named after the collector of the holotype and some paratype material; a noun in genitive case.

##### Diagnosis.

The male of this new species is similar to *S.circularis* Zhang & Li, 2011 (Zhang and Li 2011: 30, figs 8A–C, 9A–F) in having a long embolus and lacking tibial ctenidia, but it can be distinguished by the pale opisthosoma (vs yellow-brown with black markings) (Fig. 14A) and the embolus originating at the 9:00 o’clock position (vs 8:30) (Figs 13A–C, 14C–E). For the diagnosis of the female of *S.yangi*, see *S.cangshan* sp. nov. part.

##### Description.

**Male holotype** (Fig. 14A) total length 3.76. Prosoma 1.70 long, 1.42 wide; opisthosoma 2.13 long, 1.48 wide. Dorsum of prosoma brown, with high cephalic area. Eye sizes and interdistances: AME 0.07, ALE 0.11, PME 0.10, PLE, 0.11; AME–AME 0.14, AME–ALE 0.11, PME–PME 0.15, PME–PLE 0.16, ALE–PLE 0.02. MOA 0.29 long, anterior width 0.26, posterior width 0.32. Clypeus height 0.16. Chelicerae stout, brown, with 4 promarginal and 3 retromarginal teeth. Legs yellow-brown. Leg measurements: I 6.08 (1.86, 2.18, 1.28, 0.76); II 6.34 (1.72, 2.02, 1.90, 0.70); III 4.02 (1.27, 1.28, 0.93, 0.54); IV 4.48 (1.36, 1.54, 1.08, 0.50). Leg formula: 1243. Opisthosoma oval. Dorsum and venter pale. Spinnerets short and yellowish brown.

***Palp*** (Figs 13A, B, 14C–E). Tibia without ctenidia. Retrolateral tibial apophysis hook-shaped in retrolateral view. Embolus somewhat O-shaped and originating at about the 9:00 o’clock position, terminating at about the 5:00 o’clock position. Anterior arm of conductor (AA) tapering and distally reaching the 12:30 o’clock position; posterior arm (PA) spiral, with a pointed, scaly tip.

**Female paratype** (SWUC-T-DI-19-02, Fig. 14B) total length 3.78. Prosoma 1.36 long, 1.13 wide; opisthosoma 2.67 long, 1.99 wide. Dorsum of prosoma yellowish brown, with high cephalic area. Eye sizes and interdistances: AME 0.05, ALE 0.10, PME 0.09, PLE, 0.09; AME–AME 0.13, AME–ALE 0.10, PME–PME 0.14, PME–PLE 0.15, ALE–PLE 0.05. MOA 0.25 long, anterior width 0.23, posterior width 0.31. Clypeus height 0.14. Legs yellow-brown. Leg measurements: I 5.14 (1.61, 1.75, 1.07, 0.71); II 4.66 (1.52, 1.52, 1.00, 0.62); III 3.81 (1.20, 1.24, 0.85, 0.52); IV 4.58 (1.41, 1.59, 1.06, 0.52). Leg formula: 1243. Opisthosoma oval. Dorsum and venter pale. Spinnerets short and yellowish brown.

***Epigyne*** (Figs 13C, D, 14F, G). Copulatory openings large, 9-shaped, facing away from each other, separated by about 1.7 times their width. Copulatory ducts slightly sclerotized, long and abruptly bent. Spermatheca clavate, spermathecal heads round. Fertilization ducts semicircular, directed laterally.

##### Variation.

Male (*n* = 12) total length 3.76–4.15; female (*n* = 16) total length 3.78–4.57.

##### Distribution.

China (Yunnan) (Fig. 15).

## Supplementary Material

XML Treatment for Sudesna

XML Treatment for Sudesna cangshan

XML Treatment for Sudesna dali

XML Treatment for Sudesna haiboi

XML Treatment for Sudesna hainan

XML Treatment for Sudesna hedini

XML Treatment for Sudesna shangrila

XML Treatment for Sudesna yangi

## ﻿Additional information

### Author ORCIDs

Lu-Yu Wang https://orcid.org/0000-0002-5250-3473

Xian-Jin Peng https://orcid.org/0000-0002-2614-3910

Zhi-Sheng Zhang https://orcid.org/0000-0002-9304-1789

### Data availability

All of the data that support the findings of this study are available in the main text.

## References

[B1] Álvarez-Padilla F Hormiga G (2007) A protocol for digesting internal soft tissues and mounting spiders for scanning electron microscopy. Journal of Arachnology 35: 538–542. 10.1636/Sh06-55.1

[B2] Esyunin SL Sozontov AN (2016) On a new Eurasian species of *Dictyna* Sundevall, 1833 (Aranei: Dictynidae), with taxonomic notes on poorly known Palaearctic *Dictyna* species. Arthropoda Selecta 25(2): 199–206. 10.15298/arthsel.25.2.06

[B3] Feng ZQ (1990) Spiders of China in Colour. Hunan Science and Technology Publishing House, Changsha, 256 pp.

[B4] Kim ST Lee SY (2017) Arthropoda: Arachnida: Aaraneae [sic]: Oecobiidae, Oxyopidae, Cybaeidae, Dictynidae, Sparassidae, Philodromidae. Spiders II. Invertebrate Fauna of Korea 21(42): 1–122.

[B5] Lehtinen PT (1967) Classification of the cribellate spiders and some allied families, with notes on the evolution of the suborder Araneomorpha. Annales Zoologici Fennici 4: 199–468.

[B6] Marusik YM Ovtchinnikov SV Koponen S (2006) Uncommon conformation of the male palp in common Holarctic spiders belonging to the *Lathysstigmatisata* group (Araneae, Dictynidae). Bulletin of the British Arachnological Society 13(9): 353–360.

[B7] Namkung J (2002) The Spiders of Korea. Kyo-Hak Publishing, Seoul, 648 pp.

[B8] Namkung J (2003) The Spiders of Korea, 2^nd^ ed. Kyo-Hak Publishing, Seoul, 648 pp.

[B9] Paik KY (1979) Korean spiders of family Dictynidae. Research Review of Kyungpook National University 27: 419–431.

[B10] Schenkel E (1936) Schwedisch-chinesische wissenschaftliche Expedition nach den nordwestlichen Provinzen Chinas, unter Leitung von Dr. Sven Hedin und Prof. Sü Ping-chang. Araneae gesammelt vom schwedischen Arzt der Expedition Dr. David Hummel 1927–1930. Arkiv för Zoologi 29(A1): 1–314.

[B11] Song DX (1987) Spiders from agricultural regions of China (Arachnida: Araneae). Agriculture Publishing House, Beijing, 376 pp.

[B12] Song DX Lu L (1985) On some dictynids from China (Araneae: Dictynidae). Sinozoologia 3: 77–83.

[B13] Song DX Zhu MS Chen J (1999) The Spiders of China. Hebei Science and Technology Publishing House, Shijiazhuang, 640 pp.

[B14] Song DX Zhu MS Chen J (2001) The Fauna of Hebei, China: Araneae. Hebei Science and Technology Publishing House, Shijiazhuang, 510 pp.

[B15] Yin CM Bao YH Kim JP (2001) A new species of the genus *Dictyna* from China (Araneae: Dictynidae). Korean Arachnology 17: 169–172.

[B16] Yin CM Peng XJ Yan HM Bao YH Xu X Tang G Zhou QS Liu P (2012) Fauna Hunan: Araneae in Hunan, China. Hunan Science and Technology Press, Changsha, 1590 pp.

[B17] Zhang ZS Li SQ (2011) On four new canopy spiders of Dictynidae (Araneae) from Xishuangbanna rainforest, China. Zootaxa 3066: 21–36. 10.11646/zootaxa.3066.1.2

[B18] Zhang ZS Wang LY (2017) Chinese Spiders Illustrated. Chongqing University Press, Chongqing, 954 pp.

[B19] Zhu MS Shi JG (1985) Crop field spiders of Shanxi Province. Agriculture Planning Committee of Shanxi Province 1983: 239 pp.

